# Recalcitrant cutaneous sarcoidosis treated with upadacitinib: Case report

**DOI:** 10.1016/j.jdcr.2024.06.019

**Published:** 2024-07-05

**Authors:** Mohannad Safadi, Kathleen Whittington, Scott Zahner, Israel Rubinstein, Maria Tsoukas, Nadera Sweiss

**Affiliations:** a Internal Medicine Department, University of Illinois College of Medicine, Chicago, Illinois; b University of Illinois College of Medicine, Chicago, Illinois; c Aesthetic and Clinical Dermatology Associates, Hinsdale, Illinois; d Division of Pulmonary, Critical Care, Sleep, and Allergy Medicine, University of Illinois College of Medicine, Chicago, Illinois; e Department of Dermatology, University of Illinois College of Medicine, Chicago, Illinois; f Division of Rheumatology, University of Illinois College of Medicine, Chicago, Illinois

**Keywords:** cutaneous sarcoidosis, general dermatology, granulomatous inflammation, Janus kinase, lupus pernio, medical dermatology, sarcoidosisupadacitinib

## Introduction

Sarcoidosis is a multisystem chronic inflammatory disorder that is characterized by noncaseating granulomas and has an incidence of 1 to 160 per 100,000 persons depending on race and geographic location.^1^ It is most prevalent in women and begins predominately in the third and fourth decades, but evolving data indicates onset at later ages. The lungs are the most common organ affected by sarcoidosis and the skin second. Cutaneous sarcoidosis (CS) occurs in 25% to 40% of reported cases of sarcoidosis and is reported as the initial manifestation in up to 88% of cases.^1^ Black patients are more likely to have CS and at a younger age than nonblacks.^1^

The pathogenesis of sarcoidosis is still unknown but is associated with genetic, host immune, and environmental contributors.^1^^,^^2^ Recent studies have shown the involvement of 3 pathways in forming the noncaseating granulomas seen in sarcoidosis. They are the mechanistic target of rapamycin complex 1, the Janus kinase/signal transducers and activators of transcription (JAK/STAT) and the nucleotide-binding domain, leucine-rich containing family, pyrin domain-containing-3 pathways.^2^^,^^3^ These findings have led to treatment modalities for sarcoidosis including the JAK-STAT inhibitors (JAKi). To date, oral tofacitinib and topical ruxolitinib have successfully treated sarcoidosis in general and, specifically, CS.^2^^,^^4^^,^^5^ However, there are no reports of using upadacitinib (UPA) for treating CS.

## Case report

**Fig 1:**
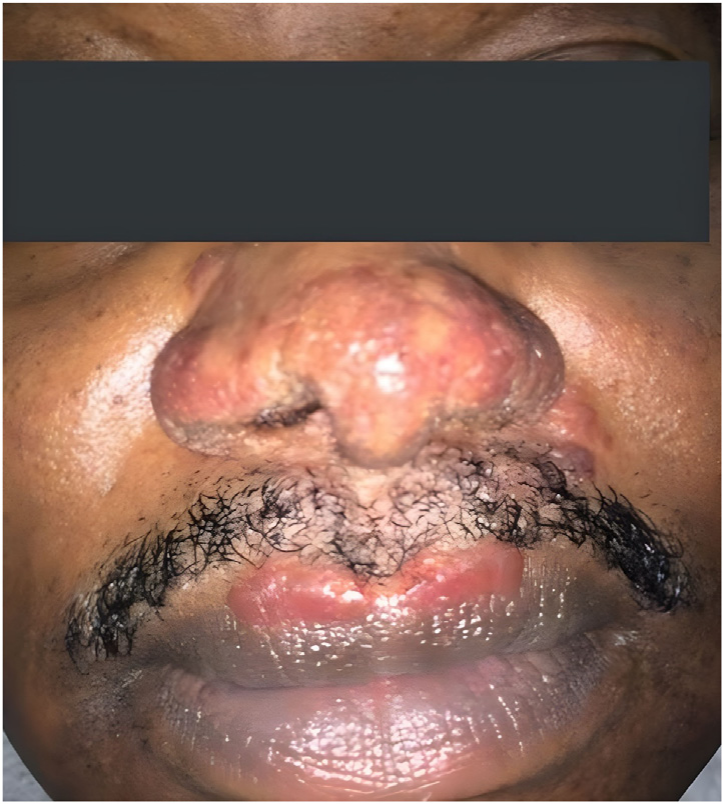
Before treatment with upadacitinib.

Our patient is a 56-year-old black man with a medical history that includes CS, psoriatic arthritis, asthma, type 2 diabetes mellitus, hypercholesterolemia, gout, hypertension, and past alcohol dependency. The patient had no signs of other system involvement of his sarcoidosis. His CS began when he was around 25 years old and started as lupus pernio presenting as shiny nodules on his nose and lips with reddish brown plaques on his head (Fig 1). Histopathology of the nose demonstrated non-necrotizing granulomas. Special stains (Gomori methenamine silver, periodic acid-Schiff and acid-fast) revealed no organisms.

Almost 30 years later, he was seen by rheumatology and dermatology clinics with persistence of his CS despite numerous pharmacotherapies including topical and systemic corticosteroids, methotrexate, infliximab, adalimumab, and repository corticotropin injections. At presentation, he was receiving the following medications: atorvastatin 20 mg, ezetimibe 10 mg, metformin 500 mg, losartan 100 mg, allopurinol 300 mg, folic acid 1 mg, montelukast 10 mg all daily. In addition, he took hydroxychloroquine 200 mg and apremilast 30 mg 2 times a day, hydrocodone-acetaminophen 5-325 1 tablet up to 4 times a day, methotrexate 15 mg subcutaneously 1 time a week and fluticasone nasal spray 2 sprays each nostril daily. The hydroxychloroquine, methotrexate and apremilast were being used for his CS and psoriatic arthritis.

**Fig 2:**
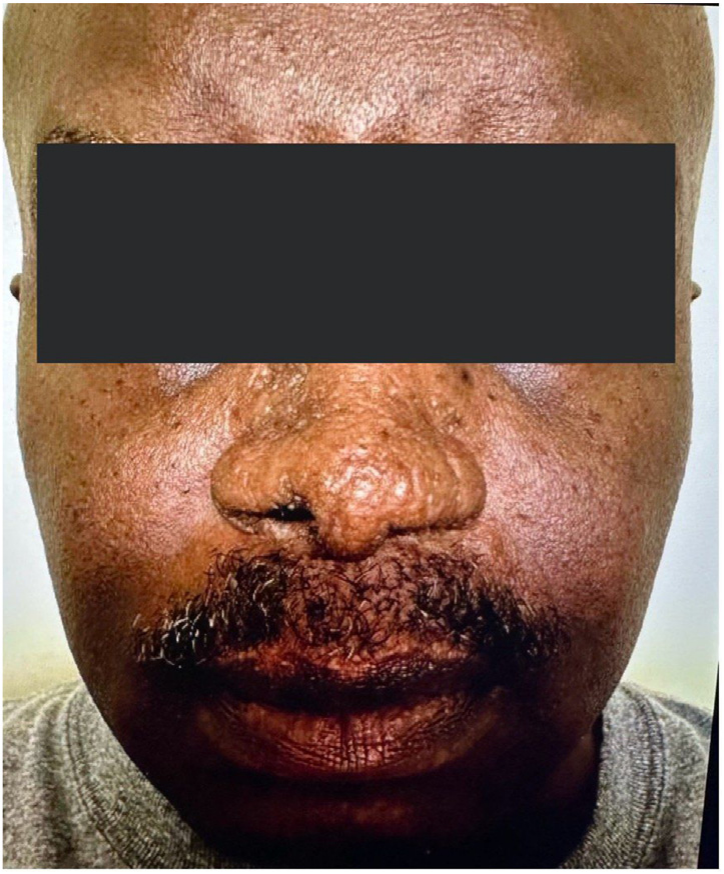
After treatment with upadacitinib.

Discussion of various treatment options by dermatology and rheumatology included restarting infliximab and the decision to try UPA for his CS given the reports of JAKi use in treating sarcoidosis. After starting UPA 15 mg daily, the patient discontinued apremilast. Six months later, the patient had an almost complete resolution of the CS lesions and improvement in his arthritis signs and symptoms (Fig 2). On further follow up, the patient discontinued his UPA and his CS lesions were returning within 6 weeks. Restarting the UPA quickly controlled the CS lesions. The patient continues to remain responsive to UPA for more than 18 months.

Consent was obtained from the patient for photographs and medical information to be published in print and online with the understanding that this information may be publicly available and will remain on file.

## Discussion

Sarcoidosis is an inflammatory disease characterized by the presence of non-necrotizing granulomas. It affects many organ systems, most commonly the lungs, skin, and lymph nodes. CS, specifically the lupus pernio type, can indicate sarcoidosis progression to other organ systems. First-line therapy for sarcoidosis involves topical, intralesional, and oral corticosteroids. To avoid adverse events of short- and long-term corticosteroids, moving to second-line medications including hydroxychloroquine, methotrexate, leflunomide, azathioprine, mycophenolate, doxycycline, or cyclophosphamide are tried to reduce those risks. Third-line medications include the use of biologics in managing CS. Reports using infliximab, adalimumab, etanercept, rituximab, golimumab, tocilizumab, apremilast and ustekinumab, the JAKi tofacitinib, ruxolitinib, and baricitinib as well as repository corticotropin injection can all be found in the literature.^6^^,^^7^

JAKi have a role in the treatment of sarcoidosis. JAK-STAT is an intracellular signaling pathway involved in many proinflammatory signaling pathways. In sarcoidosis, STAT1 is primarily activated in granuloma macrophages while activated STAT3 is in T-cells and B-cells thus indicating a role for the JAK-STAT pathway in the pathogenesis of sarcoidosis.^3^^,^^8^ Damsky et al identify type 1 cytokines, particularly interferon-gamma, as being closely correlated with disease activity and response to treatment, and the JAKi are effective modulators of the pathways involved. Several reports using JAKi topically and orally have shown effectiveness in treating sarcoidosis. Currently, the reports of JAKi in treating sarcoidosis have primarily used tofacitinib orally, a JAK1/JAK3 inhibitor, or ruxolitinib, a JAK1/JAK2 inhibitor, topically.^2^^,^^4^^,^^9^ No previous cases of sarcoidosis treated with UPA, a selective JAK1 inhibitor, have been reported.

Our patient was recalcitrant to previous medications including corticosteroids, etanercept, infliximab, adalimumab, hydroxychloroquine, methotrexate, apremilast, and repository corticotropin injection. Our patient nearly cleared after 6 months of UPA while not responding adequately to various treatments including 2 TNFi. UPA has also been shown to work in another granulomatous disease, granuloma annulare.^10^ Our findings contribute to the literature that supports the use of JAKi, in this case, UPA, in treating CS. Ascertaining which JAKi we should choose to treat sarcoidosis will need to be elucidated by further studies to see which JAKi will be most effective while having the least number of adverse events.

## Conflicts of interest

None disclosed.
